# The association between the red blood cell distribution width-to-albumin ratio and in-hospital mortality in cardiac arrest patients: an analysis of the eICU database

**DOI:** 10.3389/fcvm.2026.1838778

**Published:** 2026-07-16

**Authors:** Yuyuan Wang, Xin Yuan, Dongxia Wang, Chaoxi Liu, Ruihua Wang

**Affiliations:** 1 Department of Cardiology, Changzhi People’s Hospital, The Affiliated Hospital of Changzhi Medical College, Changzhi, Shanxi, China; 2 Department of Critical Care Medicine, Changzhi People’s Hospital, The Affiliated Hospital of Changzhi Medical College, Changzhi, Shanxi, China; 3 Department of Cardiology, Shanxi Provincial People’s Hospital, The Affiliated Hospital of Shanxi Medical University, Taiyuan, Shanxi, China

**Keywords:** albumin, cardiac arrest, eICU database, in-hospital mortality, red blood cell distribution width

## Abstract

**Background:**

Cardiac arrest triggers systemic ischemia-reperfusion injury, which initiates a cascade of inflammatory responses that particularly damage cerebral and myocardial tissues. Although the red blood cell distribution width-to-albumin ratio (RAR) has been extensively studied in various cardiovascular conditions, its prognostic value for predicting in-hospital mortality in cardiac arrest patients remains unclear. This study aimed to examine the association between RAR levels and in-hospital mortality risk in post-cardiac arrest patients.

**Methods:**

We enrolled 2,508 cardiac arrest patients admitted to intensive care units (ICUs) from the eICU Collaborative Research Database (eICU-CRD). Patients were categorized into tertiles based on RAR levels. The primary endpoint was in-hospital mortality. We employed logistic regression models, restricted cubic splines (RCS), and subgroup analyses to evaluate the association between RAR and mortality.

**Results:**

Among the 2,508 eligible patients (mean age: 63.1 years; 58.5% male), a nonlinear relationship between RAR and in-hospital mortality was identified using restricted cubic spline and threshold analyses. The association of RAR with in-hospital mortality was stronger below the RAR threshold of 5.354; at or above this cutoff, the association remained statistically significant but was markedly attenuated. Each one-unit increase in RAR was independently associated with higher in-hospital mortality risk after full adjustment (adjusted odds ratio [OR] = 1.16, 95% confidence interval [CI]: 1.10–1.22). Consistent with the continuous trend analysis, patients in the highest RAR tertile had a significantly higher mortality risk compared with those in the lowest tertile (*P* for trend <0.001). Subgroup analyses were performed for exploratory purposes. A marginally significant interaction between RAR and hypertension was observed (*P* for interaction = 0.038), suggesting a potential modifying effect of hypertension on the magnitude of the RAR–mortality association. No significant effect modification was identified for other stratified variables. Of note, the positive association between RAR and in-hospital mortality remained directionally consistent across all subgroups, despite variations in effect strength.

**Conclusion:**

In this retrospective cohort study, elevated RAR was independently associated with higher in-hospital mortality in cardiac arrest patients with a nonlinear threshold effect. While this positive association was directionally consistent across subgroups, its magnitude was significantly modified by hypertension. As a low-cost routine biomarker, RAR showed significant prognostic relevance; however, its modest predictive performance and negligible incremental value over albumin limit its clinical utility, requiring prospective external validation.

## 1 Introduction

Sudden cardiac arrest (SCA) is a life-threatening condition characterized by the abrupt cessation of cardiac function, leading to acute cardiovascular collapse and subsequent hypoperfusion of vital organs. In most cases, SCA is triggered by cardiac arrhythmias—particularly ventricular tachycardia or ventricular fibrillation—that frequently develop as complications of acute myocardial infarction (AMI) and contribute to sudden cardiac death in 25%–50% of patients within the first few hours following AMI onset (1) Despite global improvements in post-cardiac arrest survival rates over the past two decades (rising from approximately 4% in 2001 to 14% in 2020), the proportion of survivors achieving favorable neurological recovery remains suboptimal (2). Accordingly, international collaborative efforts have increasingly focused on optimizing cardiac arrest risk prediction and clinical management. For instance, the PARQ initiative aims to reduce the societal burden of SCA by elucidating its genetic, acquired, and environmental determinants and their interactions, as well as by improving the identification of high-risk individuals. For cardiac arrest survivors, systematic etiological evaluation is essential to clarify the underlying causes of unexplained SCA, thereby facilitating secondary prevention strategies and guiding individualized clinical management for patients and their families (3, 4).

Red blood cell distribution width (RDW) quantitatively reflects erythrocyte size heterogeneity, whereas serum albumin serves as a well-validated biomarker of nutritional status and systemic inflammation. The RDW-to-albumin ratio (RAR), a composite index derived from these two routine laboratory parameters, has emerged as an accessible prognostic biomarker across a wide spectrum of diseases. Accumulating evidence demonstrates that elevated RAR is independently associated with adverse clinical outcomes in multiple conditions, including malignancies (5), critical illness complicated by coronary artery disease and diabetes mellitus (6), and post-burn surgical status (7). Additionally, RAR has been correlated with diabetic retinopathy, potentially reflecting underlying systemic inflammation and oxidative stress (8, 9).

Notably, RAR exhibits robust prognostic value in cardiovascular and critical care populations. Elevated RAR predicts increased 90-day mortality in patients with acute myocardial infarction (10, 11) and reliably forecasts all-cause mortality in heart failure cohorts (12). In the field of post-cardiac arrest care, previous studies based on the MIMIC-IV database have preliminarily established an association between RAR and mortality (13, 14). However, external validation of this relationship in independent multicenter cardiac arrest cohorts remains scarce, and few studies have specifically focused on in-hospital all-cause mortality as the primary endpoint.

Against this background, the present study aimed to validate and extend these preliminary findings using the eICU Collaborative Research Database (eICU-CRD), systematically evaluating the association between admission RAR levels and in-hospital mortality among patients with in-hospital cardiac arrest.

## 2 Methods

### 2.1 Data source

Study participants were enrolled from the eICU-CRD (15), a publicly available multicenter critical care database. This database contains de-identified clinical records of over 200,000 intensive care unit (ICU) admissions from 208 hospitals across the United States between 2014 and 2015. The dataset includes comprehensive information regarding patient demographics, vital signs, laboratory results, clinical diagnoses, and therapeutic interventions, with raw data derived from bedside monitoring systems and electronic clinical documentation. The eICU-CRD is maintained by the Philips eICU Research Institute and is available to qualified researchers who complete ethical training and sign a formal data use agreement. The use of de-identified publicly available data was exempted from institutional review board (IRB) approval, and a waiver of informed consent was granted. One author obtained authorized access to the database and was responsible for all data extraction procedures.

### 2.2 Study design

**Figure 1:**
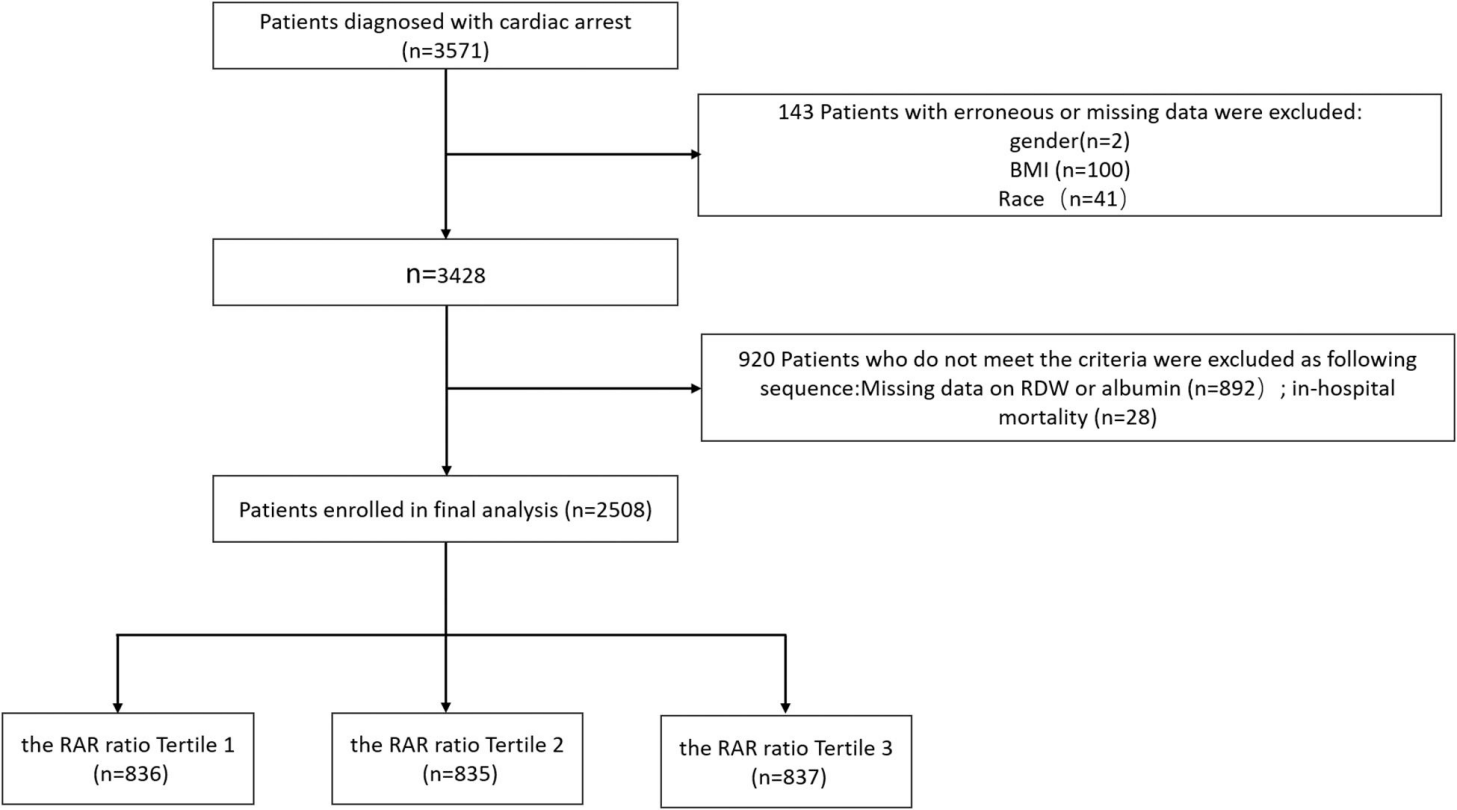
Flowchart of patient screening.

This retrospective multicenter observational cohort study analyzed de-identified clinical data from the eICU-CRD. We included only each patient's first ICU admission to avoid duplicate records from repeated hospitalizations. We screened all 200,859 ICU admissions using ICD-9 code 427.5 to identify patients with in-hospital cardiac arrest and excluded those with out-of-hospital cardiac arrest. Initial inclusion criteria were age ≥18 years and ICU length of stay ≥24 h, leaving 3,571 eligible candidates. We excluded patients with missing or biologically implausible values for core variables (sex, BMI, race, RDW, albumin, in-hospital mortality). These core variables were not imputed because missing values in the primary exposure (RDW, albumin) or the primary outcome (in-hospital mortality) would preclude a valid analysis of the RAR-mortality association irrespective of imputation; therefore, patients with missing data for any of these essential variables were excluded from the analytical cohort. Specifically, 143 patients with incomplete demographic data and 920 patients lacking valid RDW, albumin, or mortality values were excluded. The final analytical cohort consisted of 2,508 patients (Figure 1).

Detailed missing data handling strategies were standardized and clearly described in this study. Briefly, variables with missing proportions ≥20% were excluded from all analyses. For variables with missingness <20%, multiple imputation generating five complete datasets was performed to minimize information bias. Multivariate regression models were fitted on each imputed dataset, and pooled effect estimates were ultimately obtained. All statistical analyses were conducted using Free Statistics Software (version 2.5). A supplementary table summarizes all included and excluded variables, along with the exact missing percentage of each indicator. To mitigate potential selection bias resulting from the exclusion of 1,063 patients with incomplete data, a complete-case sensitivity analysis was further performed. Sensitivity analysis results were consistent with the primary analyses, confirming the robustness and reliability of our core conclusions (Supplementary Tables S1, S2).

### 2.3 Variable extraction

Data were extracted from the eICU-CRD and categorized into three modules: (1) baseline demographic and clinical characteristics, including sex, age, race (Caucasian, African American, Hispanic, Asian, Native American, and Other/Unknown), weight, and height; (2) admission vital signs: heart rate, respiratory rate, pulse oxygen saturation (SpO_2_), and body temperature; (3) laboratory biomarkers, including RDW, anion gap, alanine aminotransferase (ALT), aspartate aminotransferase (AST), alkaline phosphatase (ALP), glucose, bicarbonate, blood urea nitrogen (BUN), calcium, chloride, creatinine, hematocrit (HCT), hemoglobin (HGB), magnesium, mean corpuscular hemoglobin (MCH), mean corpuscular hemoglobin concentration (MCHC), mean corpuscular volume (MCV), partial pressure of carbon dioxide (PCO_2_), pH, platelet count (PLT), partial pressure of oxygen (PO_2_), potassium, red blood cell count (RBC), sodium, total bilirubin, total protein, and white blood cell count (WBC). All laboratory and physiological parameters represented initial values recorded within the first 24 h of ICU admission.

### 2.4 Groups and outcomes

The exposure variable RAR was calculated as RDW (%) divided by albumin (g/dL) (16). Our cohort included 2,508 patients, who were stratified into three groups by RAR tertiles: T1 (2.44–4.36, *n* = 836), T2 (4.37–5.74, *n* = 835), and T3 (5.75–23.54, *n* = 837). The primary endpoint was in-hospital mortality.

### 2.5 Statistical analysis

Continuous variables with a normal distribution are presented as mean ± SD and compared via one-way ANOVA. Skewed continuous variables are reported as median (Q1, Q3) and compared using the Kruskal–Wallis test. Categorical variables are summarized as *n* (%) and compared with the chi-square test or Fisher's exact test where appropriate.

Univariable and multivariable logistic regression models were constructed to evaluate the association between RAR and in-hospital mortality in patients with cardiac arrest. Odds ratios (ORs) and 95% confidence intervals (CIs) were computed. Covariate selection relied on Free Statistics software output combined with clinical judgment (Supplementary Table S3). Model 1 was adjusted for race, sex, age, respiratory rate, and BMI. Model 2 added adjustments for BUN, HGB, HCT, MCHC, magnesium, creatinine, potassium, sodium, RBC, and WBC on top of Model 1. Model 3 further incorporated diabetes mellitus and hypertension on the basis of Model 2.

Restricted cubic spline (RCS) regression was applied to model the nonlinear association between RAR and in-hospital mortality, and threshold analysis was performed to identify the optimal cutoff value. All restricted cubic spline curves were anchored at a reference value corresponding to an odds ratio of 1.0, which is separate from the cutoff threshold derived from segmented threshold regression. Separate logistic regression analyses were conducted for subgroups split by this threshold. ROC analyses generated AUC values for RAR, RDW, and albumin; pairwise DeLong's tests were used to compare their predictive performance. Subgroup analyses stratified by sex, age, BMI, hypertension, and diabetes were carried out to test effect modification of the RAR–mortality association across heterogeneous critically ill patients. All statistical analyses were completed using Free Statistics software (version 2.5) (16) and the R statistical package (http://www.R-project.org, The R Foundation).

## 3 Results

### 3.1 Patient and hospital characteristics

A total of 2,508 cardiac arrest patients were stratified into three tertile groups according to RAR levels (T1: 3.7 ± 0.4, *n* = 836; T2: 5.0 ± 0.4, *n* = 835; T3: 7.7 ± 2.2, *n* = 837), with significant between-group differences in mean RAR values (*P* < 0.001). Baseline comparisons showed that patients with higher RAR levels were older, more likely to be female, and had a higher prevalence of diabetes and hypertension (all *P* < 0.001); BMI also differed significantly across groups (*P* = 0.032). In terms of laboratory parameters, higher RAR was associated with elevated BUN, creatinine, serum sodium, WBC, and RDW, as well as reduced HGB, RBC, HCT, serum calcium, MCH, MCHC, MCV, and albumin (all *P* < 0.05). No significant intergroup differences were detected for serum magnesium or platelet counts (all *P* > 0.05). Notably, in-hospital mortality rose progressively from the lowest to the highest RAR tertile (33.6%, 45.9%, and 55.2%, respectively; *P* < 0.001), demonstrating that elevated RAR was positively correlated with higher in-hospital mortality among cardiac arrest patients (Table 1).

**Table 1:** Comparisons of the baseline characteristics categorized by the RAR index.

Variables	Total (*n* = 2,508)	RAR tertile 1, (*n* = 836)	RAR tertile 2, (*n* = 835)	RAR tertile 3, (*n* = 837)	*P*-value
Patient characteristics					
Sex, *n* (%)					<0.001
Female	1,041 (41.5)	272 (32.5)	369 (44.2)	400 (47.8)	
Male	1,467 (58.5)	564 (67.5)	466 (55.8)	437 (52.2)	
Age, (years)	63.1 ± 15.7	59.2 ± 16.2	64.5 ± 15.4	65.5 ± 14.8	<0.001
Race, *n* (%)					<0.001
Caucasian	1,854 (73.9)	666 (79.7)	619 (74.1)	569 (68)	
African American	347 (13.8)	80 (9.6)	118 (14.1)	149 (17.8)	
Hispanic	83 (3.3)	19 (2.3)	27 (3.2)	37 (4.4)	
Asian	16 (0.6)	6 (0.7)	4 (0.5)	6 (0.7)	
Native American	52 (2.1)	15 (1.8)	16 (1.9)	21 (2.5)	
Other/Unknown	156 (6.2)	50 (6)	51 (6.1)	55 (6.6)	
Diabetes, *n* (%)					<0.001
No	2,061 (82.2)	748 (89.5)	684 (81.9)	629 (75.1)	
Yes	447 (17.8)	88 (10.5)	151 (18.1)	208 (24.9)	
Hypertension, *n* (%)					<0.001
No	1,161 (46.3)	442 (52.9)	371 (44.4)	348 (41.6)	
Yes	1,347 (53.7)	394 (47.1)	464 (55.6)	489 (58.4)	
Vital signs					
BMI, (kg/m²)	29.8 ± 8.1	29.9 ± 7.3	30.4 ± 8.3	29.3 ± 8.7	0.032
Heart rate, (beats/min)	90.3 ± 22.9	89.3 ± 23.1	89.7 ± 23.1	92.0 ± 22.5	0.038
Respiratory rate, (breaths/min)	20.5 ± 6.6	20.3 ± 6.2	20.7 ± 6.5	20.6 ± 6.9	0.378
Temperature, (℃)	35.9 ± 1.6	35.9 ± 1.5	35.9 ± 1.5	35.9 ± 1.6	0.968
Lab values					
Albumin, (g/dL)	3.0 ± 0.7	3.7 ± 0.4	3.0 ± 0.3	2.4 ± 0.5	<0.001
BUN, (mg/dL)	22.0 (15.0, 37.0)	18.0 (14.0, 25.0)	23.0 (16.0, 35.0)	31.0 (19.0, 48.0)	<0.001
Creatinine, (mg/dL)	1.4 (1.0, 2.1)	1.2 (1.0, 1.6)	1.4 (1.0, 2.1)	1.7 (1.1, 3.0)	<0.001
Sodium, (mmol/L)	138.9 ± 5.6	136.8 ± 5.7	139.0 ± 4.8	141.0 ± 5.4	<0.001
Potassium, (mmol/L)	4.3 ± 1.0	4.1 ± 1.0	4.4 ± 1.0	4.4 ± 1.0	<0.001
RAR, (index)	5.5 ± 2.1	3.7 ± 0.4	5.0 ± 0.4	7.7 ± 2.2	<0.001
Calcium, (mmol/L)	8.5 ± 1.2	8.7 ± 1.0	8.4 ± 1.0	8.2 ± 1.4	<0.001
Magnesium, (mmol/L)	2.1 ± 0.8	2.1 ± 0.5	2.1 ± 0.6	2.0 ± 1.0	0.426
Chloride, (mmol/L)	102.3 ± 6.9	101.9 ± 6.2	102.1 ± 7.0	102.9 ± 7.4	0.005
HCT, (%)	36.5 ± 7.8	41.2 ± 6.0	36.6 ± 7.1	31.9 ± 7.3	<0.001
HGB, (g/dL)	11.9 ± 2.7	13.6 ± 2.1	11.8 ± 2.3	10.1 ± 2.3	<0.001
Platelet count, (10^9^/L)	221.3 ± 96.2	223.3 ± 73.4	222.7 ± 96.3	218.1 ± 114.5	0.481
RBC, (10^12^/L)	4.0 ± 0.9	4.4 ± 0.7	4.0 ± 0.8	3.5 ± 0.9	<0.001
WBC, (10^9^/L)	14.4 ± 7.8	14.0 ± 6.7	14.2 ± 7.5	15.0 ± 9.0	0.027
MCH, (pg)	29.8 ± 2.7	30.6 ± 2.1	29.8 ± 2.6	28.9 ± 3.2	<0.001
MCHC, (g/dL)	32.3 ± 1.6	33.0 ± 1.3	32.2 ± 1.5	31.8 ± 1.7	<0.001
MCV, (fL)	92.2 ± 7.4	92.7 ± 6.1	92.6 ± 7.1	91.2 ± 8.8	<0.001
RDW, (%)	15.4 ± 2.5	13.7 ± 1.1	15.1 ± 1.6	17.3 ± 2.9	<0.001
Patient outcomes					
In-hospital mortality, *n* (%)					<0.001
Alive	1,382 (55.1)	555 (66.4)	452 (54.1)	375 (44.8)	
Expired	1,126 (44.9)	281 (33.6)	383 (45.9)	462 (55.2)	

BMI, body mass index; BUN, blood urea nitrogen; RAR, ratio of RDW to albumin; RDW, red blood cell distribution width; HCT, hematocrit; HGB, hemoglobin; RBC, red blood cell, WBC, white blood cell; MCH, mean corpuscular hemoglobin;MCHC, mean corpuscular hemoglobin concentration; MCV, mean corpuscular volume; RDW, red blood cell distribution width.

### 3.2 Hospital all-cause mortality of cardiac arrest is associated with RAR

Univariable logistic regression was performed to identify factors associated with in-hospital mortality (Table 2). Advanced age, elevated heart rate, respiratory rate, RDW, MCV, WBC, and serum potassium, as well as RAR, were significant risk factors for in-hospital mortality, while male sex, higher body temperature, albumin, HGB, MCHC, and RBC served as protective factors (all *P* < 0.05). Race, BMI, diabetes, hypertension, and most routine biochemical and hematological indicators showed no significant association with in-hospital mortality (all *P* > 0.05).

**Table 2:** Association of covariates and in-hospital mortality.

Variable	OR (95% CI)	*P*-value
Patient characteristics		
Sex, *n* (%)		
Female	1 (reference)	
Male	0.85 (0.72–1)	0.045
Age, (years)	1.01 (1.01–1.02)	<0.001
Race, *n* (%)		
Caucasian	1 (reference)	
African American	0.93 (0.74–1.17)	0.526
Hispanic	0.84 (0.53–1.31)	0.431
Asian	0.94 (0.35–2.53)	0.897
Native American	1.64 (0.94–2.87)	0.081
Other/Unknown	0.82 (0.59–1.14)	0.231
Diabetes, *n* (%)		
	1 (reference)	
	1.06 (0.86–1.3)	0.577
Hypertension, *n* (%)		
	1 (reference)	
	0.98 (0.84–1.15)	0.825
Vital signs		
BMI, (kg/m²)	1.01 (1–1.02)	0.083
Heart rate, (beats/min)	1 (1–1.01)	0.033
Respiratory rate, (breaths/min)	1.01 (1.01,1.02)	<0.001
Temperature, (℃)	0.77 (0.73–0.82)	<0.001
Lab values		
Albumin, (g/dL)	0.58 (0.51–0.65)	<0.001
Creatinine, (mg/dL)	1.01 (0.98–1.05)	0.48
BUN, (mg/dL)	1 (1–1)	0.364
Calcium, (mmol/L)	0.99 (0.93–1.06)	0.809
Magnesium, (mmol/L)	1.03 (0.92–1.15)	0.641
RAR	1.18 (1.13–1.23)	<0.001
Sodium, (mmol/L)	1.01 (1–1.02)	0.21
Potassium, (mmol/L)	1.18 (1.09–1.28)	<0.001
Chloride, (mmol/L)	0.99 (0.98–1)	0.058
HCT, (%)	0.99 (0.98–1)	0.322
HGB, (g/dL)	0.96 (0.93–0.98)	0.003
MCH, (pg)	0.98 (0.95–1.01)	0.266
MCHC, (g/dL)	0.81 (0.77–0.85)	<0.001
MCV, (fL)	1.02 (1.01–1.03)	<0.001
RDW, (%)	1.09 (1.05–1.12)	<0.001
Platelet count, (10^9^/L)	1 (1–1)	0.273
RBC, (10^12^/L)	0.9 (0.82–0.98)	0.022
WBC, (10^9^/L)	1.02 (1.01–1.03)	0.001

BMI, body mass index; BUN, blood urea nitrogen; RAR, ratio of RDW to albumin; RDW, red blood cell distribution width; HCT, hematocrit; HGB, hemoglobin; RBC, red blood cell; WBC, white blood cell; MCH, mean corpuscular hemoglobin; MCHC, mean corpuscular hemoglobin concentration; MCV, mean corpuscular volume; RDW, red blood cell distribution width.

Multivariable logistic regression models with progressive covariate adjustment were constructed to evaluate the independent association between RAR and in-hospital mortality. Elevated RAR was significantly associated with in-hospital mortality across the crude model and three sequentially adjusted models. The crude OR for RAR was 1.18 (95% CI: 1.13–1.23, *P* < 0.001); Model 1 yielded an OR of 1.17 (95% CI: 1.13–1.22, *P* < 0.001); both Model 2 and the fully adjusted Model 3 exhibited an OR of 1.16 (95% CI: 1.10–1.22, *P* < 0.001). RAR maintained a stable positive association with in-hospital mortality after stepwise adjustment for confounding factors. Furthermore, tertile stratification analysis confirmed a significant dose–response trend across all regression models (all *P* for trend < 0.001). In the fully adjusted Model 3, relative to patients in the lowest RAR tertile (T1, reference), those in the middle tertile (T2) had a significantly higher risk of in-hospital mortality (OR = 1.48, 95% CI: 1.18–1.86, *P* = 0.001), and patients in the highest tertile (T3) showed a substantially elevated mortality risk (OR = 2.37, 95% CI: 1.82–3.09, *P* < 0.001) (Table 3).

**Table 3:** Odds ratios (95% confidence intervals) of in-hospital mortality and different RAR in different models.

Variable	Crude		Model 1		Model 2		Model 3	
	OR (95% CI)	*P*-value	OR (95% CI)	*P*-value	OR (95% CI)	*P*-value	OR (95% CI)	*P*-value
RAR	1.18 (1.13–1.23)	<0.001	1.17 (1.13–1.22)	<0.001	1.16 (1.1–1.22)	<0.001	1.16 (1.1–1.22)	<0.001
Tertile								
T1	1 (Ref)		1 (Ref)		1 (Ref)		1 (Ref)	
T2	1.67 (1.37–2.04)	<0.001	1.6 (1.3–1.95)	<0.001	1.48 (1.18–1.86)	0.001	1.48 (1.18–1.86)	0.001
T3	2.43 (2–2.97)	<0.001	2.36 (1.92–2.89)	<0.001	2.36 (1.81–3.08)	<0.001	2.37 (1.82–3.09)	<0.001
*P* for trend		<0.001		<0.001		<0.001		<0.001

Model 1 was adjusted for race, sex, age, respiratory rate, and BMI. Model 2 was further adjusted for BUN, HGB, HCT, MCHC, magnesium, creatinine, potassium, sodium, RBC, and WBC on the basis of Model 1. Model 3 was further adjusted for diabetes, hypertension on the basis of Model 2.

**Figure 2:**
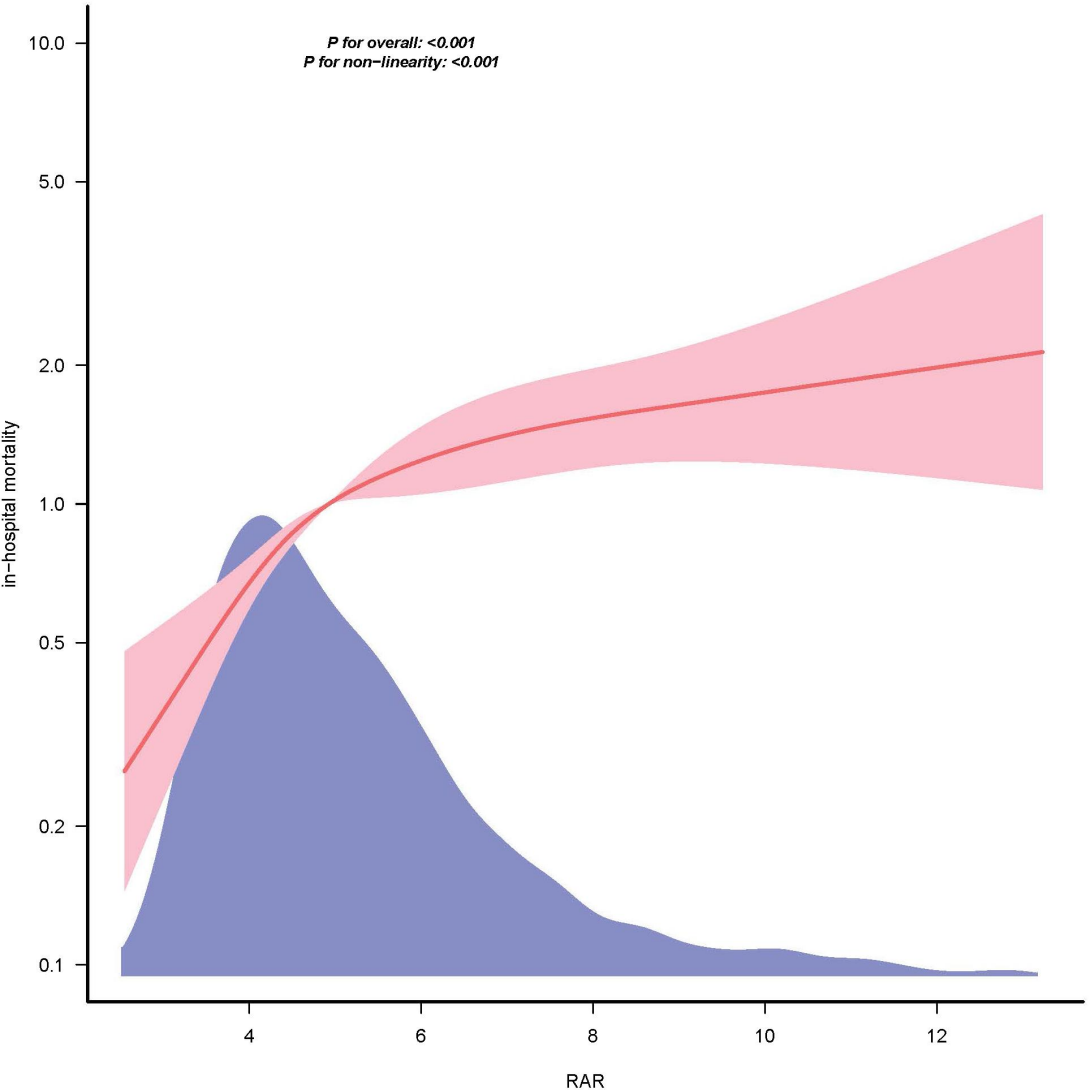
Restricted cubic spline curves for in-hospital mortality by RAR in all participants after covariate adjustment.

RCS analysis revealed a significant nonlinear relationship between RAR and in-hospital mortality among cardiac arrest patients (Figure 2). The RCS curve was anchored at a reference point with an OR of 1.0, which differs distinctly from the optimal threshold value identified via segmented regression. Segmented threshold regression identified an optimal RAR breakpoint of 5.354, indicating distinct associations across different RAR ranges. For patients with RAR values below 5.354, each one-unit increment in RAR was strongly associated with increased in-hospital mortality risk (OR = 1.554, 95% CI: 1.252–1.928, *P* < 0.001). For patients with RAR values ≥5.354, the positive association remained statistically significant but became markedly attenuated (OR = 1.102, 95% CI: 1.004–1.211, *P* = 0.0414) (Table 4).

**Table 4:** Threshold effect analysis of RAR on in-hospital mortality.

Threshold of RAR	OR	95% CI	*P* value
<5.354	1.554	1.252–1.928	<0.001
≥5.354	1.102	1.004–1.211	0.0414

Adjustment factors included race, sex, age, respiratory rate, BMI, BUN, HGB, HCT, MCHC, magnesium, creatinine, potassium, sodium, RBC, WBC, diabetes, and hypertension.

### 3.3 Subgroup analyses

**Figure 3:**
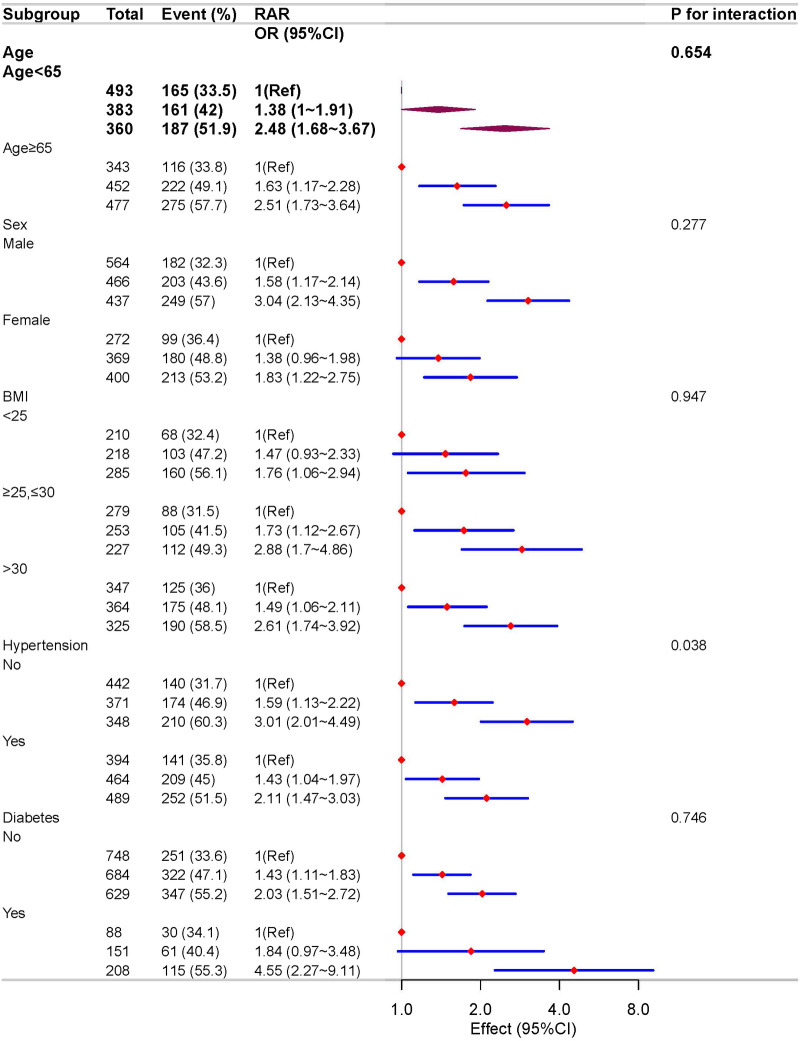
Forest plots of odds ratios for the in-hospital mortality in different subgroup. OR, odds ratio; CI, confidence interval; BMI, body mass index.

Subgroup analyses stratified by age, sex, BMI, hypertension, and diabetes were conducted in an exploratory manner, without formal correction for multiple comparisons, to examine potential effect modification of the RAR–mortality association. A marginally significant interaction was observed for hypertension (*P* for interaction = 0.038), suggesting a possible mild modifying effect on the magnitude of the prognostic association. No evidence of effect modification was detected for age (*P* = 0.654), sex (*P* = 0.277), BMI (*P* = 0.947), or diabetes (*P* = 0.746). Of note, the positive association between elevated RAR and in-hospital mortality remained directionally consistent across all stratified subgroups, albeit with minor variations in effect size. In most subgroups, only patients in the highest RAR tertile exhibited a significantly higher mortality risk compared with those in the lowest tertile (Figure 3).

### 3.4 ROC curve analysis of the RAR index

**Figure 4:**
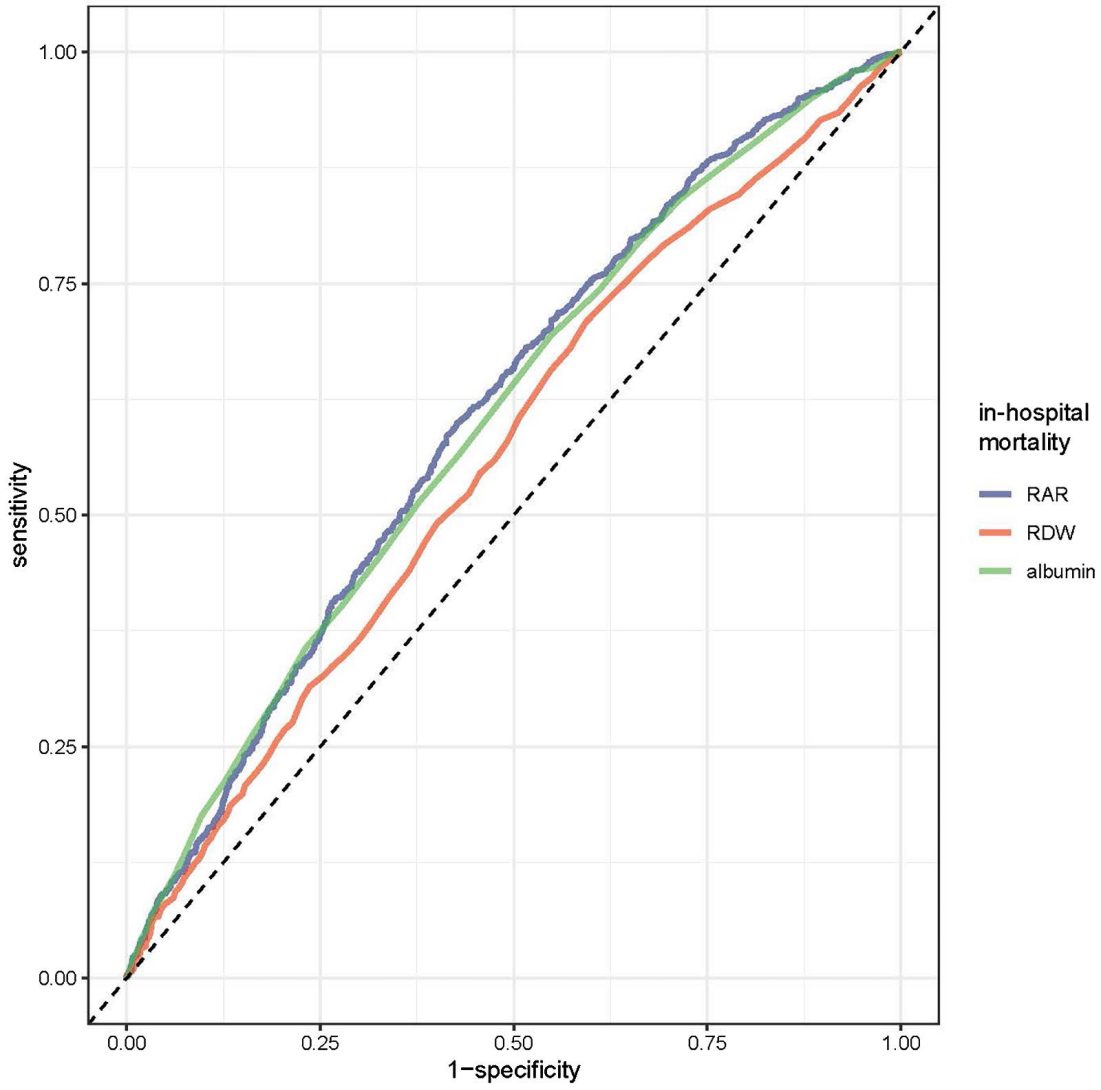
ROC curves predicting in-hospital mortality in CA patients.

ROC analyses were performed to evaluate the discriminatory ability of RAR, RDW, and albumin in predicting in-hospital mortality among patients post cardiac arrest (Figure 4). The AUC of RAR was 0.610 (95% CI: 0.588–0.632), which was statistically higher than those of RDW (AUC = 0.565, 95% CI: 0.542–0.587) and albumin (AUC = 0.603, 95% CI: 0.581–0.625). All pairwise comparisons via DeLong's test reached statistical significance (all *P* < 0.001). Notably, although RAR exhibited statistically superior discriminative capacity, its overall predictive performance remained modest. The incremental AUC improvement of RAR over albumin was minimal, indicating no robust, clinically meaningful predictive advantage.

## 4 Discussion

In this retrospective cohort study of 2,508 cardiac arrest patients from the eICU-CRD database, we demonstrated a significant positive association between elevated RAR and in-hospital mortality, which remained robust after adjusting for a comprehensive set of potential confounders, including demographics, baseline comorbidities, and key laboratory parameters. RCS analysis revealed a non-linear dose–response relationship between RAR and in-hospital mortality, with a threshold effect at an RAR value of 5.354. Subgroup analyses in the present study were exploratory and not adjusted for multiple comparisons. A marginally significant interaction indicated that hypertension may slightly modify the prognostic magnitude of RAR for in-hospital mortality (*P* for interaction = 0.038). However, this finding should be interpreted cautiously due to the risk of false positivity from multiple subgroup testing and should not be overinterpreted. No significant effect modification was observed across age, sex, BMI, and diabetes subgroups. Overall, the positive prognostic association of RAR was qualitatively stable across different clinical characteristics, supporting the general robustness of RAR as a prognostic biomarker in cardiac arrest populations.

Our results align with and extend prior evidence on the prognostic value of RAR in critically ill populations. Cai et al. recently reported that elevated RAR was independently associated with 30-day and 180-day all-cause mortality in post-cardiac arrest patients from the MIMIC-IV database (13), which aligns with our finding of a robust association between RAR and in-hospital mortality in this high-risk cohort. However, to our knowledge, this study is among the first to systematically characterize the nonlinear dose–response relationship and threshold effect of RAR in cardiac arrest patients. Identify its threshold effect, and explore potential subgroup effect modification across multiple clinically relevant variables, including a marginally significant interaction related to hypertension. Previous studies have separately established that elevated RDW (a marker of erythropoietic dysfunction, chronic inflammation, and oxidative stress) and reduced albumin (a marker of malnutrition, systemic inflammation, and hepatic dysfunction) are independent predictors of poor prognosis in patients with cardiovascular disease and critical illness (17). As a composite index integrating both nutritional and inflammatory status, RAR reflects cumulative inflammatory and nutritional derangements; our fully adjusted analyses demonstrate its prognostic value beyond individual RDW or albumin levels after accounting for potential confounders.

The biological mechanisms underlying the association between elevated RAR and increased in-hospital mortality in cardiac arrest patients are likely multifactorial. First, cardiac arrest and subsequent resuscitation trigger systemic ischemia–reperfusion injury and a profound systemic inflammatory response (SIRS), which can suppress erythropoiesis and increase RDW, while simultaneously promoting capillary leakage, hepatic dysfunction, and reduced albumin synthesis (18, 19). Second, chronic inflammation and malnutrition—reflected by elevated RDW and reduced albumin—may correlate with clinical frailty (20). Frailty, characterized by diminished physiological reserve (i.e., a reduced capacity to withstand acute physiological stress) (21), is known to worsen tolerance to post-arrest ischemic injury and increase the risk of multi-organ dysfunction, including myocardial failure, acute kidney injury, and severe neurological damage (22). The non-linear association between RAR and in-hospital mortality was further validated by threshold analysis, which identified an optimal breakpoint at a RAR value of 5.354. Specifically, in patients with RAR <5.354, each one-unit increase in RAR was strongly predictive of elevated in-hospital mortality, with a steeply graded risk increment (OR = 1.554, 95% CI: 1.252–1.928, *P* < 0.001). Although the positive association persisted among individuals with RAR ≥5.354, the predictive effect was substantially attenuated and marginally significant (OR = 1.102, 95% CI: 1.004–1.211, *P* = 0.0414). At low RAR levels, subtle perturbations in systemic inflammation and nutritional status may disrupt physiological homeostasis, impairing hepatic albumin synthesis and erythropoietic function and thereby gradually increasing mortality risk. In contrast, further RAR elevation beyond the 5.354 threshold yields only a modest additional prognostic impact, indicating a diminished marginal risk effect at higher RAR levels. Notably, the correlation between RAR and physiological frailty remains indirect. Given that RAR reflects the combined burden of inflammation and malnutrition, its association with reduced physiological reserve is inferred from existing evidence, rather than directly verified by dedicated frailty assessment tools. Further basic and translational investigations are warranted to clarify the exact biological mechanisms underlying the relationship between RAR and post-cardiac arrest survival (23).

Several limitations exist in this retrospective multicenter observational study. First, key cardiac arrest prognostic factors and CPC functional scores were missing from the eICU-CRD, leading to unmeasured confounding and inability to evaluate long-term neurological outcomes. Second, only admission RAR was analyzed, without assessing dynamic RAR changes during ICU stay. Third, the all-adult US in-hospital cardiac arrest cohort restricts generalizability to other populations, requiring external validation. Fourth, logistic regression for binary mortality cannot compare death timing across RAR groups. Fifth, excluding patients with missing core variables may cause mild selection bias, despite similar baseline features between included and excluded participants. Lastly, this observational study only demonstrates associations, not causality; prospective studies are needed to explore mechanisms linking RAR to poor prognosis.

Our analyses showed that RAR, an inexpensive routine composite laboratory marker, was independently associated with in-hospital mortality among cardiac arrest patients. Its AUC of 0.610 marginally exceeded that of albumin (0.603), with no meaningful incremental predictive value for clinical use. Therefore, we cannot confirm that incorporating RAR into prognostic models improves risk stratification or guides targeted interventions based solely on RAR. Prospective external validation is required to replicate this association. Further studies should investigate whether interventions targeting RAR-related inflammation and malnutrition improve survival, as well as evaluate the relationships between RAR and long-term neurological and functional outcomes.

## 5 Conclusions

This retrospective cohort analysis revealed that elevated RAR was independently associated with higher in-hospital mortality among cardiac arrest patients, with an evident nonlinear threshold effect. While the positive association between RAR and mortality remained directionally consistent across subgroups, hypertension significantly altered its effect magnitude. As an inexpensive, routinely measurable biomarker, RAR yielded statistically significant prognostic relevance. Nevertheless, RAR demonstrated only modest discriminatory ability and minimal incremental predictive value compared with albumin, restricting its clinical utility and necessitating further prospective external validation.

## Data Availability

The data analyzed in this study is subject to the following licenses/restrictions: access to the dataset requires prior approval from the eICU Research Institute, and the data may only be used for non-commercial academic research purposes. The dataset shall not be reproduced, distributed, or modified without official authorization, and all research results using this dataset must appropriately cite the eICU database as the data source. Additionally, researchers are required to comply with relevant ethical guidelines and ensure that no personally identifiable information of patients is disclosed during data analysis and manuscript writing. Requests to access these datasets should be directed to Yuyuan Wang 3060462039@qq.com.
