# Supplementary material for: The association between the red blood cell distribution width-to-albumin ratio and in-hospital mortality in cardiac arrest patients: an analysis of the eICU database
Source: Front Cardiovasc Med. 2026 Jul 16;13:1838778. doi: 10.3389/fcvm.2026.1838778 (PMC13422551; doi:10.3389/fcvm.2026.1838778)
Supplement: Supplementary file 1 [file Table1.docx]

**1、All variables**

| **No** | **Variable** | **Stats / Values** | **Freqs (% of Valid)** | **Valid** | **Missing** | **Method** |
| --- | --- | --- | --- | --- | --- | --- |
| **1** | **age\  [numeric]** | **Mean (sd) : 63.5 (15.5)\  min < med < max:\  18 < 65 < 90\  IQR (CV) : 21 (0.2)** | **73 distinct values** | **3571\  (100.0%)** | **0\  (0.0%)** | **/** |
| **2** | **gender\  [factor]** | **1\. 0\  2\. 1** | **\1478 (41.4%)\  \2091 (58.6%)** | **3569\  (99.9%)** | **2\  (0.1%)** | **Exclude missing data** |
| **3** | **BMI\  [numeric]** | **Mean (sd) : 33 (120)\  min < med < max:\  0 < 28.4 < 5524.2\  IQR (CV) : 9.5 (3.6)** | **3096 distinct values** | **3537\  (99.0%)** | **34\  (1.0%)** | **Exclude missing data** |
| **4** | **height\  [numeric]** | **Mean (sd) : 169.6 (12.8)\  min < med < max:\  0 < 170.2 < 236.2\  IQR (CV) : 15.2 (0.1)** | **165 distinct values** | **3538\  (99.1%)** | **33\  (0.9%)** | **Exclude missing data** |
| **5** | **race\  [factor]** | **1\. 1\  2\. 2\  3\. 3\  4\. 4\  5\. 5\  6\. 6** | **\2602 (73.8%)\  \ 507 (14.4%)\  \ 129 ( 3.7%)\  \ 22 ( 0.6%)\  \ 64 ( 1.8%)\  \ 202 ( 5.7%)** | **3526\  (98.7%)** | **45\  (1.3%)** | **Exclude missing data** |
| **6** | **weight\  [numeric]** | **Mean (sd) : 86.7 (28.3)\  min < med < max:\  0 < 82.1 < 771.2\  IQR (CV) : 30.7 (0.3)** | **1017 distinct values** | **3506\  (98.2%)** | **65\  (1.8%)** | **Exclude missing data** |
| **7** | **heartrate\  [character]** | **1\. NULL\  2\. 80\  3\. 90\  4\. 86\  5\. 88\  6\. 84\  7\. 70\  8\. 77\  9\. 85\  10\. 75\  [ 125 others ]** | **\ 223 ( 6.2%)\  \ 88 ( 2.5%)\  \ 80 ( 2.2%)\  \ 69 ( 1.9%)\  \ 67 ( 1.9%)\  \ 64 ( 1.8%)\  \ 63 ( 1.8%)\  \ 62 ( 1.7%)\  \ 59 ( 1.7%)\  \ 57 ( 1.6%)\  \2739 (76.7%)** | **3571\  (100.0%)** | **0\  (0.0%)** | **/** |
| **8** | **nibp_diastolic\  [numeric]** | **Mean (sd) : 69.8 (20.9)\  min < med < max:\  2 < 68 < 185\  IQR (CV) : 26 (0.3)** | **137 distinct values** | **3287\  (92.0%)** | **284\  (8.0%)** | **Imputation** |
| **9** | **nibp_systolic\  [numeric]** | **Mean (sd) : 120.5 (30.1)\  min < med < max:\  31 < 117 < 247\  IQR (CV) : 38 (0.2)** | **182 distinct values** | **3287\  (92.0%)** | **284\  (8.0%)** | **Imputation** |
| **10** | **respiratoryrate\  [numeric]** | **Mean (sd) : 20.4 (6.7)\  min < med < max:\  0 < 19 < 59\  IQR (CV) : 8 (0.3)** | **57 distinct values** | **3275\  (91.7%)** | **296\  (8.3%)** | **Imputation** |
| **11** | **spo2\  [numeric]** | **Mean (sd) : 96.4 (7.6)\  min < med < max:\  0 < 99 < 100\  IQR (CV) : 4 (0.1)** | **55 distinct values** | **2932\  (82.1%)** | **639\  (17.9%)** | **Imputation** |
| **12** | **temperature\  [numeric]** | **Mean (sd) : 36 (1.5)\  min < med < max:\  26.4 < 36.4 < 44.3\  IQR (CV) : 1.6 (0)** | **177 distinct values** | **3446\  (96.5%)** | **125\  (3.5%)** | **Imputation** |
| **13** | **albumin\  [numeric]** | **Mean (sd) : 3 (0.7)\  min < med < max:\  0.7 < 3.1 < 5.3\  IQR (CV) : 0.9 (0.2)** | **43 distinct values** | **2870\  (80.4%)** | **701\  (19.6%)** | **Exclude missing data** |
| **14** | **alkalinephos\  [numeric]** | **Mean (sd) : 111.4 (88.1)\  min < med < max:\  16 < 90 < 1374\  IQR (CV) : 58 (0.8)** | **325 distinct values** | **2825\  (79.1%)** | **746\  (20.9%)** | **Exclusion** |
| **15** | **alt\  [numeric]** | **Mean (sd) : 202.8 (612.9)\  min < med < max:\  4 < 57 < 11980\  IQR (CV) : 112 (3)** | **574 distinct values** | **2828\  (79.2%)** | **743\  (20.8%)** | **Exclusion** |
| **16** | **ast\  [numeric]** | **Mean (sd) : 331.5 (1157.9)\  min < med < max:\  4 < 83 < 24920\  IQR (CV) : 160 (3.5)** | **669 distinct values** | **2839\  (79.5%)** | **732\  (20.5%)** | **Exclusion** |
| **17** | **basos\  [numeric]** | **Mean (sd) : 0.3 (0.4)\  min < med < max:\  0 < 0.1 < 3\  IQR (CV) : 0.4 (1.5)** | **24 distinct values** | **2321\  (65.0%)** | **1250\  (35.0%)** | **Exclusion** |
| **18** | **bedglucose\  [numeric]** | **Mean (sd) : 191 (93)\  min < med < max:\  13 < 168 < 624\  IQR (CV) : 114 (0.5)** | **432 distinct values** | **2743\  (76.8%)** | **828\  (23.2%)** | **Exclusion** |
| **19** | **bun\  [numeric]** | **Mean (sd) : 29.5 (22.7)\  min < med < max:\  3 < 22 < 254\  IQR (CV) : 21 (0.8)** | **155 distinct values** | **3524\  (98.7%)** | **47\  (1.3%)** | **Imputation** |
| **20** | **calcium\  [numeric]** | **Mean (sd) : 8.4 (1.2)\  min < med < max:\  1.2 < 8.4 < 23.5\  IQR (CV) : 1.1 (0.1)** | **100 distinct values** | **3463\  (97.0%)** | **108\  (3.0%)** | **Imputation** |
| **21** | **chloride\  [numeric]** | **Mean (sd) : 102.6 (6.9)\  min < med < max:\  65 < 103 < 134\  IQR (CV) : 8 (0.1)** | **80 distinct values** | **3513\  (98.4%)** | **58\  (1.6%)** | **Imputation** |
| **22** | **creatinine\  [numeric]** | **Mean (sd) : 2 (2)\  min < med < max:\  0.1 < 1.3 < 22.1\  IQR (CV) : 1.1 (1)** | **592 distinct values** | **3530\  (98.9%)** | **41\  (1.1%)** | **Imputation** |
| **23** | **crp\  [numeric]** | **Mean (sd) : 173.3 (632.4)\  min < med < max:\  0.2 < 7.5 < 4569\  IQR (CV) : 17.6 (3.6)** | **66 distinct values** | **75\  (2.1%)** | **3496\  (97.9%)** | **Exclusion** |
| **24** | **crphs\  [factor]** | **1\. 0.5\  2\. 2.6\  3\. 7.1\  4\. 14.87\  5\. 15.73\  6\. 54.6\  7\. 55.02\  8\. 193.49** | **\1 (12.5%)\  \1 (12.5%)\  \1 (12.5%)\  \1 (12.5%)\  \1 (12.5%)\  \1 (12.5%)\  \1 (12.5%)\  \1 (12.5%)** | **8\  (0.2%)** | **3563\  (99.8%)** | **Exclusion** |
| **25** | **crphs_uom\  [factor]** | **1\. mg/L** | **\8 (100.0%)** | **8\  (0.2%)** | **3563\  (99.8%)** | **Exclusion** |
| **26** | **directbilirubin\  [numeric]** | **Mean (sd) : 0.5 (1.1)\  min < med < max:\  0 < 0.2 < 18.1\  IQR (CV) : 0.3 (2.2)** | **73 distinct values** | **800\  (22.4%)** | **2771\  (77.6%)** | **Exclusion** |
| **27** | **eos\  [numeric]** | **Mean (sd) : 1.1 (2.5)\  min < med < max:\  0 < 0.5 < 100\  IQR (CV) : 1.5 (2.3)** | **76 distinct values** | **2451\  (68.6%)** | **1120\  (31.4%)** | **Exclusion** |
| **28** | **fibrinogen\  [numeric]** | **Mean (sd) : 322.3 (159.5)\  min < med < max:\  22 < 309.5 < 979\  IQR (CV) : 208.2 (0.5)** | **299 distinct values** | **448\  (12.5%)** | **3123\  (87.5%)** | **Exclusion** |
| **29** | **hct\  [numeric]** | **Mean (sd) : 36.3 (7.8)\  min < med < max:\  12.3 < 36.7 < 70.9\  IQR (CV) : 11.1 (0.2)** | **388 distinct values** | **3513\  (98.4%)** | **58\  (1.6%)** | **Imputation** |
| **30** | **hdl\  [numeric]** | **Mean (sd) : 40.5 (16.6)\  min < med < max:\  4 < 38 < 143\  IQR (CV) : 17 (0.4)** | **79 distinct values** | **610\  (17.1%)** | **2961\  (82.9%)** | **Exclusion** |
| **31** | **hgb\  [numeric]** | **Mean (sd) : 11.8 (2.7)\  min < med < max:\  4 < 11.8 < 23.7\  IQR (CV) : 3.9 (0.2)** | **154 distinct values** | **3500\  (98.0%)** | **71\  (2.0%)** | **Imputation** |
| **32** | **ldl\  [numeric]** | **Mean (sd) : 76.5 (36.1)\  min < med < max:\  2 < 71 < 188\  IQR (CV) : 48 (0.5)** | **143 distinct values** | **483\  (13.5%)** | **3088\  (86.5%)** | **Exclusion** |
| **33** | **lymphocytes\  [numeric]** | **Mean (sd) : 19.2 (16.9)\  min < med < max:\  0 < 13 < 99\  IQR (CV) : 21.3 (0.9)** | **442 distinct values** | **2606\  (73.0%)** | **965\  (27.0%)** | **Exclusion** |
| **34** | **magnesium\  [numeric]** | **Mean (sd) : 2 (0.7)\  min < med < max:\  0.6 < 2 < 26.1\  IQR (CV) : 0.6 (0.3)** | **118 distinct values** | **3034\  (85.0%)** | **537\  (15.0%)** | **Imputation** |
| **35** | **mch\  [numeric]** | **Mean (sd) : 29.8 (2.8)\  min < med < max:\  13.9 < 30 < 46\  IQR (CV) : 3 (0.1)** | **187 distinct values** | **3203\  (89.7%)** | **368\  (10.3%)** | **Imputation** |
| **36** | **mchc\  [numeric]** | **Mean (sd) : 32.4 (1.6)\  min < med < max:\  25.4 < 32.5 < 37.8\  IQR (CV) : 2.1 (0.1)** | **111 distinct values** | **3383\  (94.7%)** | **188\  (5.3%)** | **Imputation** |
| **37** | **mcv\  [numeric]** | **Mean (sd) : 92 (7.4)\  min < med < max:\  58.6 < 92 < 136\  IQR (CV) : 8.4 (0.1)** | **372 distinct values** | **3385\  (94.8%)** | **186\  (5.2%)** | **Imputation** |
| **38** | **monos\  [numeric]** | **Mean (sd) : 6.2 (3.5)\  min < med < max:\  0 < 6 < 62.7\  IQR (CV) : 4 (0.6)** | **158 distinct values** | **2584\  (72.4%)** | **987\  (27.6%)** | **Exclusion** |
| **39** | **mpv\  [numeric]** | **Mean (sd) : 9.8 (1.4)\  min < med < max:\  5.9 < 9.8 < 14.1\  IQR (CV) : 1.9 (0.1)** | **80 distinct values** | **2399\  (67.2%)** | **1172\  (32.8%)** | **Exclusion** |
| **40** | **phosphate\  [numeric]** | **Mean (sd) : 4.7 (2.4)\  min < med < max:\  0.3 < 4.1 < 24\  IQR (CV) : 2.8 (0.5)** | **137 distinct values** | **2204\  (61.7%)** | **1367\  (38.3%)** | **Exclusion** |
| **41** | **plateletcount\  [numeric]** | **Mean (sd) : 219.7 (95.5)\  min < med < max:\  4 < 209 < 924\  IQR (CV) : 114 (0.4)** | **481 distinct values** | **3466\  (97.1%)** | **105\  (2.9%)** | **Imputation** |
| **42** | **polys\  [numeric]** | **Mean (sd) : 70.7 (19.2)\  min < med < max:\  1 < 77 < 98\  IQR (CV) : 25.4 (0.3)** | **467 distinct values** | **2276\  (63.7%)** | **1295\  (36.3%)** | **Exclusion** |
| **43** | **potassium\  [numeric]** | **Mean (sd) : 4.3 (1)\  min < med < max:\  1.3 < 4.2 < 9.7\  IQR (CV) : 1.1 (0.2)** | **101 distinct values** | **3521\  (98.6%)** | **50\  (1.4%)** | **Imputation** |
| **44** | **prealbumin\  [numeric]** | **Mean (sd) : 15.3 (6.4)\  min < med < max:\  3 < 14 < 35\  IQR (CV) : 9.6 (0.4)** | **53 distinct values** | **123\  (3.4%)** | **3448\  (96.6%)** | **Exclusion** |
| **45** | **pt\  [numeric]** | **Mean (sd) : 17.7 (10)\  min < med < max:\  9 < 14.9 < 229.2\  IQR (CV) : 5.2 (0.6)** | **344 distinct values** | **2702\  (75.7%)** | **869\  (24.3%)** | **Exclusion** |
| **46** | **ptinr\  [numeric]** | **Mean (sd) : 1.6 (1)\  min < med < max:\  0.8 < 1.2 < 14.1\  IQR (CV) : 0.4 (0.6)** | **244 distinct values** | **2791\  (78.2%)** | **780\  (21.8%)** | **Exclusion** |
| **47** | **ptt\  [numeric]** | **Mean (sd) : 40.2 (24.8)\  min < med < max:\  16 < 32.8 < 269\  IQR (CV) : 14.2 (0.6)** | **502 distinct values** | **2326\  (65.1%)** | **1245\  (34.9%)** | **Exclusion** |
| **48** | **pttratio\  [factor]** | **1\. FALSE\  2\. TRUE** | **\18 (24.7%)\  \55 (75.3%)** | **73\  (2.0%)** | **3498\  (98.0%)** | **Exclusion** |
| **49** | **rbc\  [numeric]** | **Mean (sd) : 4 (0.9)\  min < med < max:\  1.2 < 4 < 7.5\  IQR (CV) : 1.2 (0.2)** | **427 distinct values** | **3483\  (97.5%)** | **88\  (2.5%)** | **Imputation** |
| **50** | **rdw\  [numeric]** | **Mean (sd) : 15.3 (2.4)\  min < med < max:\  11.6 < 14.7 < 33.2\  IQR (CV) : 2.8 (0.2)** | **143 distinct values** | **3261\  (91.3%)** | **310\  (8.7%)** | **Exclude missing data** |
| **51** | **sodium\  [numeric]** | **Mean (sd) : 138.3 (5.7)\  min < med < max:\  98 < 139 < 173\  IQR (CV) : 7 (0)** | **83 distinct values** | **3530\  (98.9%)** | **41\  (1.1%)** | **Imputation** |
| **52** | **tc\  [numeric]** | **Mean (sd) : 141.8 (43.5)\  min < med < max:\  44 < 137 < 297\  IQR (CV) : 59 (0.3)** | **173 distinct values** | **600\  (16.8%)** | **2971\  (83.2%)** | **Exclusion** |
| **53** | **totalbilirubin\  [numeric]** | **Mean (sd) : 0.9 (1.6)\  min < med < max:\  0 < 0.6 < 33.2\  IQR (CV) : 0.5 (1.7)** | **129 distinct values** | **2734\  (76.6%)** | **837\  (23.4%)** | **Exclusion** |
| **54** | **totalprotein\  [numeric]** | **Mean (sd) : 6.1 (1)\  min < med < max:\  2 < 6.2 < 11.4\  IQR (CV) : 1.3 (0.2)** | **76 distinct values** | **2819\  (78.9%)** | **752\  (21.1%)** | **Exclusion** |
| **55** | **triglycerides\  [numeric]** | **Mean (sd) : 143.9 (111.2)\  min < med < max:\  17 < 113 < 1519\  IQR (CV) : 94.2 (0.8)** | **273 distinct values** | **784\  (22.0%)** | **2787\  (78.0%)** | **Exclusion** |
| **56** | **uricacid\  [numeric]** | **Mean (sd) : 9.1 (5.7)\  min < med < max:\  1.4 < 8.4 < 41.3\  IQR (CV) : 3.9 (0.6)** | **37 distinct values** | **45\  (1.3%)** | **3526\  (98.7%)** | **Exclusion** |
| **57** | **wbc\  [numeric]** | **Mean (sd) : 14.4 (7.9)\  min < med < max:\  0.1 < 13 < 111.1\  IQR (CV) : 8.7 (0.6)** | **908 distinct values** | **3502\  (98.1%)** | **69\  (1.9%)** | **Imputation** |
| **58** | **hospdischargestatus\  [factor]** | **1\. 0\  2\. 1** | **\2028 (57.3%)\  \1510 (42.7%)** | **3538\  (99.1%)** | **33\  (0.9%)** | **Imputation** |
| **59** | **hosplosday\  [numeric]** | **Mean (sd) : 10.6 (21.2)\  min < med < max:\  0.4 < 6.8 < 763.5\  IQR (CV) : 9.6 (2)** | **1736 distinct values** | **3571\  (100.0%)** | **0\  (0.0%)** | **/** |
| **60** | **unitdischargestatus\  [factor]** | **1\. 0\  2\. 1** | **\2399 (67.2%)\  \1171 (32.8%)** | **3570\  (100.0%)** | **1\  (0.0%)** | **/** |
| **61** | **unitlosday\  [numeric]** | **Mean (sd) : 6 (6.9)\  min < med < max:\  1 < 3.8 < 108.4\  IQR (CV) : 4.9 (1.2)** | **1239 distinct values** | **3571\  (100.0%)** | **0\  (0.0%)** | **/** |
| **62** | **diabetes\  [factor]** | **1\. 0\  2\. 1** | **\2945 (82.5%)\  \ 626 (17.5%)** | **3571\  (100.0%)** | **0\  (0.0%)** | **/** |
| **63** | **hypertension\  [factor]** | **1\. 0\  2\. 1** | **\1627 (45.6%)\  \1944 (54.4%)** | **3571\  (100.0%)** | **0\  (0.0%)** | **/** |
|  |  |  |  |  |  |  |
|  |  |  |  |  |  |  |
|  |  |  |  |  |  |  |

2、**Table S2. Under the complete case analysis,Odds ratios (95% confidence intervals) of in-hospital mortality and different RAR in different models.**

 Model 1 was adjusted for race, gender, age, respiratory rate, and BMI. Model 2 was further adjusted for BUN, HGB, HCT, MCHC, magnesium, creatinine, potassium, sodium, RBC, and WBC on the basis of Model 1. Model 3 was further adjusted for diabetes, hypertension on the basis of Model 2.

**3、Table S3 Covariate screening table**
